# Type B Lactic Acidosis Secondary to Metastatic Liver Cancer in the Setting of Normal Renal Function: A Case Report and Literature Review

**DOI:** 10.7759/cureus.61583

**Published:** 2024-06-03

**Authors:** Chidinma Amakiri, Tracy-Ann Poyser, Steven Pham, Blake Alwardt, Collie Shaw

**Affiliations:** 1 Internal Medicine, Unity Health, White County Medical Center, Searcy, USA

**Keywords:** oncological emergency, metastatic liver cancer, malignancy, continuous renal replacement therapy (crrt), metabolic acidosis, lactic acidosis

## Abstract

Lactic acidosis occurs from an overproduction of lactate or decreased metabolism. It is common in critically ill patients, especially those with hematological conditions such as multiple myeloma, leukemia, and lymphoma. There are two types of lactic acidosis, Type A and Type B, with Type B presenting more commonly in hematological conditions that require prompt diagnosis and treatment of the underlying condition. We present a case of a 43-year-old male with Type B lactic acidosis secondary to stage IV colon cancer with metastasis to the liver. Initial laboratory work was significant for lactic acid of 16.52 mmol/L. Arterial blood gas (ABG) showed pH 7.26, pCO_2_ 21 mmHg, pO_2_ 111 mmHg, and HCO_3_ 9 mEq/L, revealing an anion gap and metabolic acidosis with compensatory respiratory alkalosis. Initially, the patient was treated with aggressive fluid management, IV antibiotics, and sodium bicarbonate; however, his lactic acid continued to rise. The recommendation was made for urgent dialysis. Despite treatments, the prognosis is poor.

## Introduction

Lactate levels can be elevated in conditions that result in increased lactate production and/or decreased metabolism and clearance [1]. Lactic acidosis occurs when the pH is less than 7.35 and blood lactate levels are greater than 5mmol/L [2]. It is produced from glucose and glycogen and then metabolized to carbon dioxide and water or to glucose [1]. The liver is the primary site of lactic acid clearance where it is converted back to glucose via the lactic acid cycle, followed by the kidneys [3]. Liver failure is thus an etiology for lactic acidosis. The buildup of lactic acid results in increased hydrogen ions and decreased generation of bicarbonate [4,5]. An accumulation of lactic acid can be the result of poor tissue oxygenation, referred to as type A lactic acidosis, or in the setting of normal oxygenation without identifiable impairment, also known as type B lactic acidosis, and lastly with intestinal bacterial proliferation also known as type D lactic acidosis [6]. Lactic acidosis is a common cause of metabolic acidosis with and without an anion gap in severely ill hospitalized patients [4,7]. Type B lactic acidosis occurs due to toxins or tissue hypoperfusion [1]. Common causes include diabetes mellitus, alcohol poisoning, infection, mitochondrial disorders, beta-adrenergic agonists, and rarely, malignancy [1]. The etiology of lactic acidosis in patients with solid cancers is uncertain but may be attributed to decreased lactic clearance [7]. Type B lactic acidosis is an oncologic emergency that has a poor prognosis without definitive treatment of the underlying cause [4,5]. Type D is seen in patients with gastrointestinal abnormalities affecting absorption such as short bowel syndrome or ischemic bowel disease [6].

## Case presentation

We report a case of a 43-year-old Caucasian male with a history of stage IV colon cancer metastasizing to the liver, who presented to the emergency department for worsening shortness of breath and generalized weakness. He initially went to the medical room at his prison unit for evaluation where his lab work resulted as abnormal. He was taken to the emergency department at an outside facility for further workup. At the emergency department, Computed tomography (CT) of the chest showed evidence of pneumonia in the right lower lobe. Initial laboratory work was significant for lactic acid of 16.52 mmol/L. ABG showed pH 7.26, pCO_2_ 21 mmHg, pO_2_ 111 mmHg, and HCO_3_ 9 mEq/L, revealing an anion gap metabolic acidosis with compensatory respiratory alkalosis. He had a glucose level of 52 mg/dL and bicarbonate of 8 mEq/L at the outside facility. He was started on intravenous (IV) Vancomycin 2 gram every 12 hours, IV Cefepime 1 gram every 8 hours, 50 mEq of sodium bicarbonate, 1 liter bolus of normal saline, and 25 gram of D50 prior to transfer under the care of our hospitalist in our facility. 

On arrival at our facility, the patient was found to be afebrile with a blood pressure of 143/88 mmHg, pulse of 90 beats per minute, respiratory rate of 18 breaths per minute, and oxygen saturation of 99% on room air. On examination, he was found to have tenderness to palpation in the right upper quadrant and normal respiratory effort with clear breath sounds bilaterally. The remainder of the physical examination was unremarkable. Laboratory testing, shown in Table 1, revealed an elevated serum lactate of 13.6 mmol/L. Bicarbonate is low at 7 mEq/L.

**Table 1:** Laboratory data demonstrating the abnormalities (with a *) during hospitalization *Lab value lower than the normal range; **Lab value higher than the normal range ALT = alanine transaminase; AST = aspartate aminotransferase; ALP = alkaline phosphatase; BUN = blood urea nitrogen; Hgb = hemoglobin; Hct = hematocrit; Plt = platelet; WBC = white blood cell

Pertinent labs (Normal range)	On admission	Day 2	Day 3	Day 4	Day 5	Day 6	Day 7	Day 8
Sodium (136-145 mEq/L)	132^*^	134^*^	134^*^	133^*^	132^*^	129^*^	134^*^	134^*^
Potassium (3.5-5 mEq/L)	3.9	3.6	3.8	3.7	3.8	3.7	3.6	3.3
Anion gap (7-13 mEq/L)	25^**^	24^**^	27^**^	25^**^	29^**^	28^**^	27^**^	29^**^
Bicarbonate (23-28 mEq/L)	7^*^	8^*^	6^*^	5^*^	6^*^	5^*^	5^*^	<5^*^
BUN (7-20 mg/dL)	12	10	8^*^	11	10	7	10	15
Creatinine (0.6-1.3 mg/dL)	0.7	0.7	0.6	0.6	0.7	0.6	0.8	1.1
AST (10-40 u/L)	203^**^	252^**^	187^**^	165^**^		186^**^	163^**^	131^**^
ALT (10-40 u/L)	252^**^	152^**^	119^**^	113^**^		113^**^	112^**^	94^**^
ALP (30-120 u/L)	923^**^	1045^**^	837^**^	876^**^		1015^**^	1006^**^	881^**^
Lactic acid (0.7-2.1 mmol/L)	13.6^**^	16.7^**^	18.9^**^	17.8^**^	18.4^**^	19.4^**^	18.8^**^	23.4^**^
WBC (4.5-11 k/uL)	7.9	8.3	6.7	8.8	8.9		11.3	13.7^**^
Hgb (14-18 g/dL)	11.7*	12.2*	12.4*	10.4*	10.7*		10.5*	10.1*
Hct (42-50%)	38.6*	40.2*	40.2*	34.6*	35.2*		34.5*	33.4*
Plt (150-450 k/uL)	199	194	125*	151	178		176	139*
Venous pH (7.32-7.41)	7.23	7.19			7.24			
Venous CO2 (42-53 mmHg)	25.3	20.4			22.9			
pH			7.22					
pCO2			16.7					
pO2			124.0					
HCO3			6.6					

**Figure 1:**
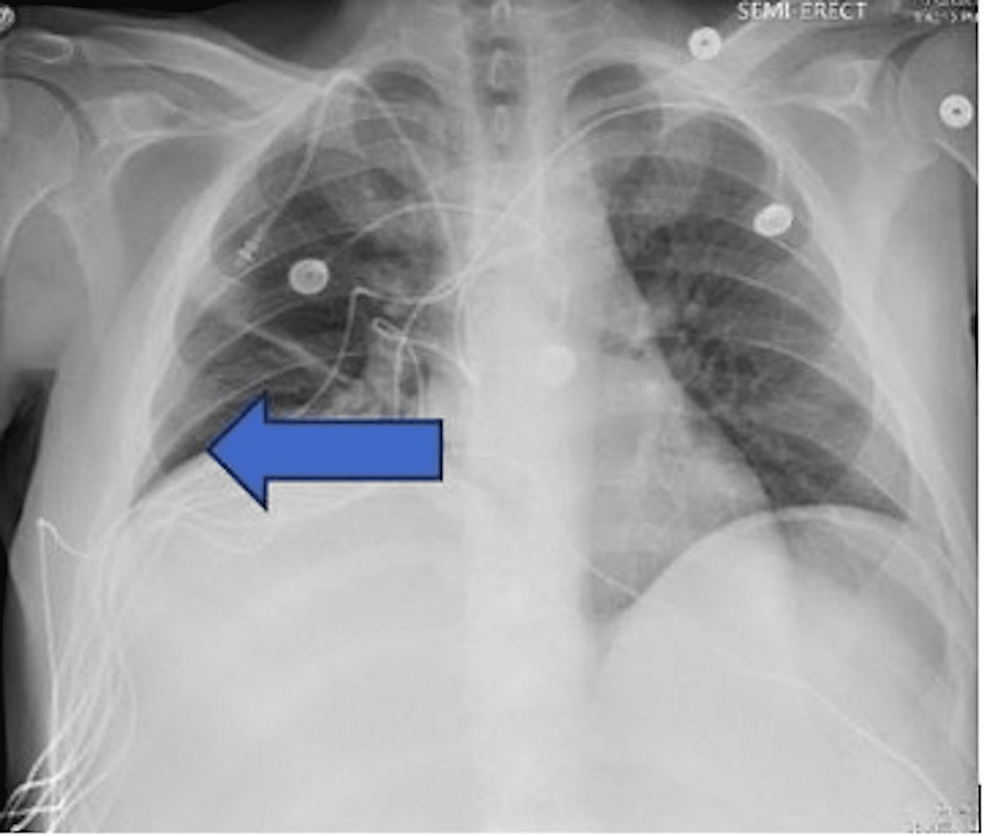
Chest X-ray showing elevated right hemidiaphragm secondary to liver burden. Atelectasis/scarring of the right lung base is noted by the blue arrow.

**Figure 2:**
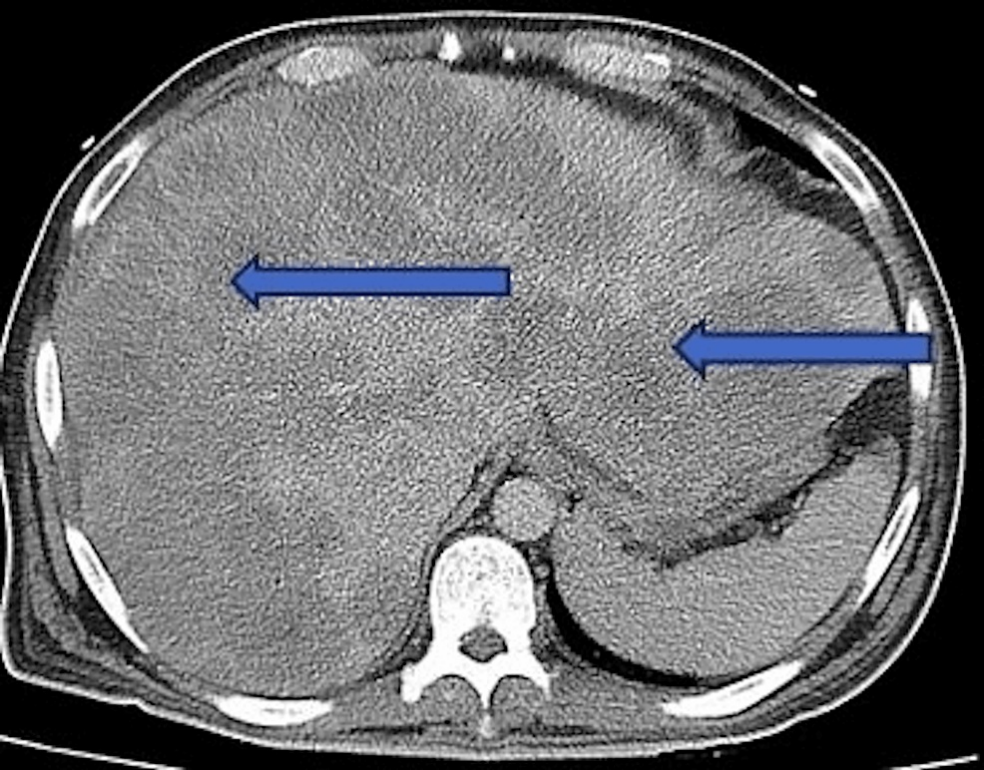
CT abdomen/pelvis showing extensive liver metastasis with significant hepatomegaly noted by the blue arrows. Liver measured greater than 30 cm in length. Splenomegaly and ascites were also noted in the study.

Initial chest X-ray, as shown in Figure 1, demonstrated atelectasis, and scarring on the right lung base (blue arrow). CT of the abdomen and pelvis with contrast demonstrated extensive hepatic metastatic disease with marked hepatomegaly, splenomegaly, and mild ascites as shown in Figure 2.

He was diagnosed with a severe anion gap metabolic acidosis, secondary to type B lactic acidosis from stage IV colon cancer with liver metastasis. Additionally, he was diagnosed with sepsis without shock secondary to pneumonia.

The patient was empirically treated with broad-spectrum IV antibiotics. Vancomycin and cefepime were dosed as above, and metronidazole was started at 500 milligrams every 8 hours. He was started on a bicarbonate drip and oral bicarbonate tablets due to severe acidosis. Blood cultures were obtained, which resulted in no growth. Basic metabolic panel and lactic acid levels were trended. Vancomycin and cefepime were later discontinued in favor of doxycycline and piperacillin/tazobactam.

Throughout his hospitalization, he continued to complain of dyspnea and abdominal pain. Despite discussions about his imaging and the extent of his liver metastasis, he expressed that he wanted aggressive medical treatment. Therefore, Nephrology and General Surgery were consulted. The surgeon noted that he was not a good surgical candidate for resection due to his lactic acidosis and poor liver function. Nephrology recommended urgent dialysis to resolve his severe metabolic acidosis in the setting of lactic acidosis that had not improved with bicarbonate supplementation. He had some marginal improvement in his lactic acid levels (Table 1) following his first session of dialysis on hospital Day 4; however, it was only temporizing. After two consecutive sessions of dialysis, no significant improvement was noted in his anion gap, lactic acid levels, and bicarbonate levels or his symptoms. Additionally, dialysis was not continued to prevent a metabolic alkalosis on top of his existing acidosis. On his last day at our hospital, his anion gap was widened at 29 mEq/L while bicarbonate levels continued to be worsened to < 5 mEq/L and lactic acid increased to 23.4 mmol/L. Additionally, he was hypoglycemic despite being on D5W with bicarbonate. Oncologists believed his prognosis was poor and that he would not be a good candidate for chemotherapy due to the high risk of complications and his advanced stage. Eventually, palliative care was consulted to provide him with pain control and establish hospice care once he was discharged back to prison.

## Discussion

Type B lactic acidosis can arise as a complication of solid and more commonly, hematological malignancies such as multiple myeloma, lymphoma, and leukemia [8]. The underlying mechanism of this process is secondary to anaerobic glycolysis [9]. Tumor cells have a high glucose turnover rate in comparison to non-cancerous cells [9]. Yet, these cells produce lactate via anaerobic metabolism [9]. We describe a case of severe anion gap metabolic acidosis, secondary to type B lactic acidosis due to stage IV colon cancer with liver metastasis. Our patient initially presented with worsening dyspnea, generalized weakness, and pneumonia. His laboratory results were significant for elevated lactic acid levels, high anion gap metabolic acidosis, low bicarbonate levels, hypoglycemia, and elevated transaminases. This was secondary to poor lactic clearance, secondary to his liver metastasis from stage IV colon cancer.

Lactic acidosis is classified into three types: type A, type B, and type D [6]. Type A is present in the setting of hypoxia and poor tissue perfusion [4]. Type B occurs in normal perfusion states and can be seen in certain underlying conditions such as diabetes mellitus, liver disease, thiamine deficiency, seizures, and mitochondrial toxins [1]. Type D is seen with excess D-lactic acid in intestinal bacterial proliferation in patients with gastrointestinal abnormalities affecting absorption [6]. The mechanism of type B lactic acidosis in patients with cancer involves the counterbalance of the Cori cycle in the liver [9]. In patients with cancer, particularly liver cancer, there is a decreased clearance of lactate, which results in the production of lactic acidosis due to the impairment of gluconeogenesis [9]. This, in turn, causes an impairment of the gluconeogenic pathway and creates a metabolic acidosis picture [9].

The most common form of lactic acidosis in patients with cancer is Type B lactic acidosis, which is associated with hematological malignancies [2]. This was observed in 87% of hematological cases and at least 13% in non-hematological cases while liver involvement was observed in 45% [10]. In our case, liver involvement seen in this patient was conducted through a CT scan of the abdomen, which confirmed the diagnosis of liver metastasis with significant hepatomegaly, splenomegaly, and multiple hypodense nodules.

Based on the literature review, the treatment options for type B lactic acidosis are not fully established [3,4]. The presenting symptoms of the cases we reviewed were non-specific and related to the underlying condition or malignancy. The gold standard to improve patient outcomes is to treat the underlying condition [2,4,11]. However, treatment modalities, such as bicarbonate therapy, hemodialysis, and chemotherapy, were studied [4,10,12]. Hemodialysis helps remove excess lactic acid from the bloodstream. Our patient received two sessions of hemodialysis in addition to sodium bicarbonate supplementation and glucose supplementation with mild initial improvement in his symptoms and later worsening of his lactic acidosis. Chemotherapy was not initiated, as it was deemed non-beneficial due to the late stage of his cancer. However, studies have shown that supplementing glucose could worsen lactic acidosis [2,8]. It was inconclusive whether his lactic acidosis may have worsened because of glucose supplementation or due to the natural course of his condition. Regardless, the reviewed studies demonstrated that patients with lactic acidosis in the setting of underlying malignancy had poor outcomes and survival.

## Conclusions

Type B lactic acidosis in patients with ongoing hematological conditions needs to be promptly identified. Underlying conditions need to be timely addressed to improve outcomes and minimize complications, though the prognosis remains poor. Due to the rarity of cases of severe acidosis, there is a paucity of available information regarding appropriate therapeutic approaches, which makes the management of this condition challenging regardless of the available treatment. To improve the outcome of these conditions, there should be a better understanding of the pathogenesis and further improvement of our modalities to effectively treat the underlying disease.
